# Morphine for elective endotracheal intubation in neonates: a randomized trial [ISRCTN43546373]

**DOI:** 10.1186/1471-2431-4-20

**Published:** 2004-10-05

**Authors:** Brigitte Lemyre, Joanne Doucette, Angela Kalyn, Shari Gray, Michael L Marrin

**Affiliations:** 1 Department of Pediatrics, Division of Neonatology, University of Ottawa, Ottawa, Canada; 2 Department of Pediatrics, Division of Newborn Medicine, Hamilton Health Sciences Corporation, McMaster University Medical Center, Hamilton, Canada; 3 Department of Pharmacy, Hamilton Health Sciences Corporation, McMaster University Medical Center, Hamilton, Canada

## Abstract

**Background:**

Elective endotracheal intubations are still commonly performed without premedication in many institutions. The hypothesis tested in this study was that morphine given prior to elective intubations in neonates would decrease fluctuations in vital signs, shorten the duration of intubation and reduce the number of attempts.

**Methods:**

From December 1999 to September 2000, infants of all gestations admitted to a level III neonatal intensive care unit and requiring an elective endotracheal intubation were randomly assigned to receive morphine 0.2 mg/kg IV or placebo 5 minutes before intubation. Duration of severe hypoxemia (HR< 90/min and Sp0_2_<85%), duration of procedure, duration of hypoxemia (Sp0_2_<85%), number of attempts and change in mean blood pressure were compared between groups.

**Results:**

34 infants (median 989 g and 28 weeks gestation) were included. The duration of severe hypoxemia was similar between groups. Duration of procedure, duration of hypoxemia, number of attempts and increases in mean blood pressure were also similar between groups. 94% of infants experienced bradycardia during the procedure.

**Conclusion:**

We failed to demonstrate the effectiveness of morphine in reducing the physiological instability or time needed to perform elective intubations. Alternatives, perhaps with more rapid onset of action, should be considered.

## Background

Endotracheal intubation is a painful and stressful procedure, which is associated with acute increases in blood pressure and intracranial pressure, bradycardia and hypoxemia [1]. These physiologic changes are potentially of sufficient magnitude to produce the reperfusion injury and venous congestion associated with intraventricular hemorrhage (IVH) and periventricular leukomalacia (PVL) [2,3]. It has been clearly demonstrated that newborn infants feel pain. More so, premature infants likely have an increased sensitivity to pain [4], which can lead to chronic pain or neurobehavioral and developmental sequelae [5,6].

Most premature infants and many term infants admitted to neonatal intensive care units (NICU) will require one or more endotracheal intubations during their stay. In 1994, 84% of Canadian NICUs, including ours, rarely or never used premedication for elective intubations [7]. In 2000, the majority of units used premedication 50–75% of the time in infants greater than 30 weeks gestation, but only rarely in those 30 weeks gestation or less [8]. Perceived lack of evidence of benefits and fear of side effects were reasons.

A literature review revealed six randomized controlled trials [9-14], comparing various combinations of premedications, which have enrolled one hundred and thirty newborn infants. Bradycardia can be ameliorated by atropine [9,10] or glycopyrrolate [11]. Increases in intracranial pressure can be dampened by muscle relaxants [9-12]. Analgesics, which seem warranted, have been minimally studied alone [13], but seem to limit the increase in blood pressure when combined with muscle relaxants [11]. A recent metaanalysis concludes that overall, premedication appears beneficial, either in stabilizing vital signs or decreasing the duration of the procedure, but data is limited about which medications are best to achieve optimal conditions [15].

We reviewed our policy, which did not include premedication for elective endotracheal intubations, in light of the current evidence. As morphine has been used for years in neonates with apparent safety and efficacy for pain and as staff in our unit were comfortable with this medication, we aimed to evaluate the efficacy of morphine, in achieving better intubation conditions and success while maintaining vital signs stability.

## Methods

### Study population

Infants of all gestations, admitted to McMaster University Medical Center level III NICU and considered likely to need an elective oral or nasotracheal intubation during their hospital stay, were candidates for inclusion in this study. Families were approached for consent as soon as possible after birth when an elective intubation during their hospital stay seemed likely: if their infant(s) was less than 30 weeks gestation, already ventilated (as endotracheal tubes are frequently changed after 10 days if clinical deterioration from a respiratory standpoint), was on NCPAP for respiratory distress or was needing an elective surgery. Others were approached when an elective intubation was needed. At the time of this study, our unit was a 33-bed level 3 NICU, caring for both inborn and outborn patients, and the referral center for 25000 annual deliveries, with 900–1000 admissions per year.

Infants were excluded if they met any of the following conditions: 1) absence of an intravenous access, 2) upper airway anomaly potentially leading to a difficult intubation, 3) cyanotic heart disease, 4) upper gastrointestinal obstruction (which would require a rapid sequence intubation) or 5) concurrent opioid administration.

### Study intervention

Infants requiring an elective intubation were randomly assigned to receive either morphine 0.2 mg/kg IV or placebo (0.9% NaCl), given over 1 minute, followed 5 minutes later by the intubation. This larger dose of morphine was chosen for the perceived acuity of pain produced by an intubation; a larger dose may be more effective to decrease the struggling by infants during the procedure, which is caused by pain. Infants were randomized according to a computer-generated random number table with random block sizes. Morphine and placebo were supplied in identical unidose vials, labeled PIN Rx, which were prepared by one pharmacist according to the randomization sequence and placed in sealed, consecutively numbered envelopes, which were opened just before intubation. Thus, randomization occurred just prior to intubation.

Three to four minutes after receiving the study medication, infants were preoxygenated with 100% 0_2 _and hand-ventilated with a self-inflatable bag for 1–2 minutes prior to intubation. Infants having their endotracheal tube replaced were ventilated through their existing tube until it was removed. Vital signs (HR, BP, Sp0_2_) were captured to a laptop computer from the infant's monitor (PC Express, Spacelabs Inc., Redmond WA) every 5 seconds (except blood pressure which was obtained with a self inflating cuff every minute) using Procom Plus Communication Software, from the time the study medication was given (which was considered the baseline) to 5 minutes after the infant's vital signs returned to pre-procedure values. One of three investigators, not involved in the procedure collected the following data manually: duration of the procedure (defined as the time between insertion of the laryngoscope in the mouth to confirmation of endotracheal tube placement by auscultation) and the number of intubation attempts (defined as number of times the laryngoscope was inserted in the mouth). If there was more than one attempt, the clock continued between attempts and was stopped only when tube placement was confirmed by auscultation. In our NICU, the preferred method of intubation is via the nasotracheal route if mechanical ventilation is expected for more than a few hours.

All team members performed the intubations: staff neonatologists ^a^, neonatal fellows ^b^, pediatric residents ^c^, clinical nurse specialists ^d^, clinical nurse specialist students ^e ^and transport nurses ^f^. After 2 unsuccessful attempts by a junior team member (c,d,e,f), a more experienced intubator (a,b) was called.

Institutional ethics committee approval and informed consent from the parents were obtained for this study.

### Outcome measures

The study aimed to test the hypothesis that morphine 0.2 mg/kg would decrease fluctuations in vital signs, shorten the duration of the procedure and reduce the number of attempts. The primary outcome was the duration of severe hypoxemia, defined as Sp0_2 _< 85% with a HR< 90/min. This was felt to be the most undesirable side effect of endotracheal intubation as cerebral blood flow in neonates is highly dependent upon heart rate. Secondary outcomes included: (1) duration of the procedure, (2) duration of hypoxemia (Sp0_2 _< 85%), (3) number of attempts, (4) maximum change in blood pressure from baseline, (5) occurrence of bradycardia (HR<90/min).

### Sample size

The study group's impression was that a majority of infants experience some degree of severe hypoxemia during an elective intubation, which was clinically undesirable. It was estimated to be 30 seconds, based on experience. In order to detect a one standard deviation difference in duration of severe hypoxemia between the 2 groups (α = 0.05, 2-sided, β = 0.2), 17 patients per group were required.

### Statistical analysis

Because the distribution of the main outcome was skewed and groups were small, continuous variables were compared using the Mann-Whitney U test. Dichotomous variables were compared using Fisher's exact test or Chi-square test. A p value < 0.05 (2-sided) was considered significant for the primary outcome; p < 0.01 was considered significant for secondary outcomes to account for multiple analyses in a small sample. Level of experience of the intubator, birth weight and gestational age were separately explored as potential confounders of the primary outcome using ANOVA or linear regression.

## Results

**Figure 1:**
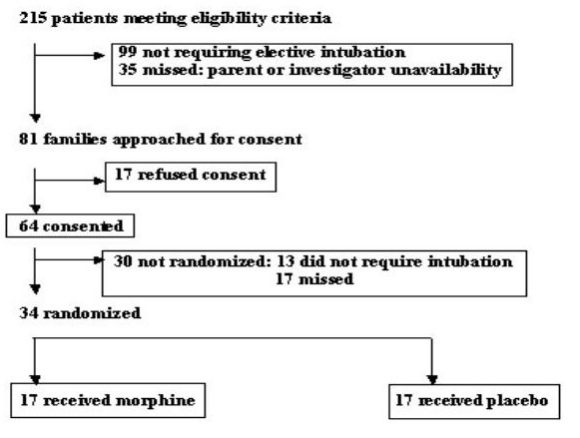
Flow of patients at each stage of the study

Patients were recruited from December 1999 to September 2000. Patient flow in the study is depicted in figure 1. Two hundred and fifteen infants were identified as potential candidates for the study. Ninety-nine of them never required an elective intubation but 35 did, they were missed, as parents or investigators were not available at the time. Eighty-one families were approached for consent. Consent was obtained for 64 infants of whom 34 were enrolled and randomized. Thirty were not randomized: 13 never required an intubation and 17 elective intubations were missed, mainly because they happened at night, when investigators were not on site. Missed patients had similar gestation, birth weight and reason for intubation as those enrolled. All patients randomized received the intervention and data from all randomized patients were analyzed. Physiological stability was maintained in all infants, between the time the study drug was given, to the time the endotracheal intubation was performed.

Baseline characteristics are presented in Table 1. Both groups had similar birth weight, gestation, and baseline vital signs. Importantly, the number of primary intubations/failed extubation and changes of endotracheal tube (which is usually considered easier) were similar between groups. All intubations were nasotracheal. Results of the primary and secondary outcomes analysis are presented in Table 2. Only 8/17 infants in the treatment group and 7/17 in the control group experienced some degree of severe hypoxemia. The median duration of severe hypoxemia was similar between groups. An outlier (severe hypoxemia lasting for 300 seconds) was identified in the treatment group. Considering the small number of patients, this outlier was taken out and the data reanalyzed, but this did not change the results significantly. The level of experience of the intubator, birth weight and gestation were entered separately in a regression model, but none was a significant contributor to the variance of the results.

**Table 1:** Baseline characteristics of included patients *Values expressed as medians (interquartile range)

	**Morphine group n = 17**	**Control group n = 17**
Birth weight, grams*	1065 (731.5, 2043)	904 (689, 1535.5)
Gestation, weeks*	28 (26, 33)	27 (26, 30)
Gender male/female, n	11/6	9/8
Age at randomization, days*	3 (0.61, 16)	8 (0.63, 13)
Primary intubation/failed extubation, n	7	7
Change of endotracheal tube, n	10	10
Baseline HR, bpm*	152 (142.5, 157.5)	161 (151.5, 166.5)
Baseline Sp0_2, _%*	94 (92.5, 95)	94 (92.5, 98)
Baseline BP, mm Hg*	38.5 (35.25, 44.75)	37.5 (33.25, 44.5)
Baseline fi0_2, _%*	40 (27, 45)	32 (25, 41.25)
Experienced intubator, n	9	7
Junior intubator, n	8	10

**Table 2:** Primary and secondary outcomes results *Values expressed as medians and interquartile range

	**Morphine group**	**Control group**	**p value**
Duration of severe hypoxemia, seconds*	10 (0, 62.5)	5 (0, 45)	0.45
Duration of hypoxemia, seconds*	235 (82.5, 340)	90 (20, 187.5)	0.04
Duration of procedure, seconds*	271 (57.5, 418.5)	94 (62, 215.5)	0.27
Maximum increase in mean BP from baseline, mm Hg*	18 (9, 24.25)	20 (11.75, 28)	0.65
Number of attempts, n*	2 (1, 3.5)	1 (1, 2.5)	0.34
Intubation achieved at first attempt, n	7	9	0.49
Intubation needing rescue intubator, n	7	4	0.27
Bradycardia during procedure, n	16	12	0.175

All patients in the treatment group and 14/17 in the control group experienced hypoxemia (Sp0_2 _< 85%) during intubation. The median duration of hypoxemia was 235 sec in the treatment group and 90 sec in the control group (p = 0.04). Because of our small sample and the likelihood of finding a significant result by chance alone when assessing multiple outcomes, it was decided a priori that a p value of 0.01 would be considered significant for secondary outcomes. Nevertheless, this represents an interesting but somewhat worrisome trend. No difference was found in the maximal increase in blood pressure. Ninety-four percent of patients experienced bradycardia (HR<90/min) during the procedure with no difference between groups.

The median duration of the procedure was 271 sec in the treatment group and 94 sec in the control, which was not statistically significant. Roughly half of the infants required more than one attempt to achieve successful intubation and the clock was not stopped between attempts. Number of attempts in the treatment group (median 2), was similar to controls (median 1); total number of attempts was 38 in the premedicated infants versus 31 in the controls. Success rate at first attempt or need to call a more senior intubator after 2 failed attempts did not differ between groups. Because of the higher than usual dose of morphine that was used, we monitored the need to increase ventilator support over the next 24 h, in infants having their tubes changed, but found no difference between groups.

## Discussion

Newborn infants, especially premature ones have adverse physiological responses to routine care procedures [2,4]. Endotracheal intubation is a stressful procedure, associated with physiologic instability [9-14]. Our data also show this instability, with 94% of infants experiencing bradycardia and the mean blood pressure increasing by as much as 46%.

Our hypothesis was that a moderate dose of morphine would facilitate intubation and stabilize vital signs better than placebo. Our data does not support this hypothesis. No significant difference was identified between the treatment and the control group in any prespecified outcome. The choice of severe hypoxemia as the primary outcome, although clinically very important, significantly limited the number of observations and increased the possibility of a type 2 error, as few infants met the criteria defining this outcome. The onset of action of morphine is about 5 minutes in infants, but the peak action occurs only at 15 to 30 minutes [16], perhaps too long for a procedure such as an intubation, as it does not lead to sufficient relaxation to permit adequate airway visualization. Although there was no formal assessment of the level of sedation of our infants done, bedside nurses reported not being able to discriminate between groups 5 minutes after injection of the study drug.

The only trend we identified was the duration of hypoxemia, which appeared longer in the treatment group. Most desaturations were in the mid 70's to low 80's range, but this is still a worrisome finding. We were unable to identify if birth weight, gestation or experience level of the intubator were significant contributors. Our sample size likely did not permit to identify such a contributor. Although morphine may not be potent enough to significantly relax infants to permit quicker and easier intubations, it may lead to decreased functional residual capacity in partially sedated infants, which could account for prolonged desaturations. The hypoxemia could have been compounded by the use of self-inflating bag and masks, which cannot provide a positive end-expiratory pressure (PEEP). Also, the larger dose of morphine used in this study could have contributed to this potential problem, by further decreasing the FRC in partially sedated infants. This trend is in keeping with the finding that, although not statistically significant, median duration of the procedure was 3 times as long in the treatment group as in the controls.

Ninety four percent of infants experienced bradycardia, mostly vagal, during their intubation. As cerebral blood flow in infants is greatly dependent on heart rate, our data adds to the current knowledge that including atropine in the premedication appears warranted. Although there may be concern that atropine could mask hypoxia-induced bradycardia, the now universal use of oxygen saturation monitors should ensure that hypoxemia is identified.

Previous trials have used various combinations of drugs for premedication and overall, they suggest that premedication is effective and safe. Kelly [9] and Barrington [10], using atropine and a muscle relaxant, demonstrated a reduction in vagal bradycardia and a dampening in the rise in intracranial pressure. The use of a muscle relaxant without an analgesic would now be considered unacceptable practice. Friesen [12] compared atropine alone to atropine and a non-standardized anesthetic and pancuronium in stable term infants preoperatively. The treatment group had less increase in intracranial pressure. This study included only stable infants. Pokela [11] compared 10 infants randomly allocated to glycopyrrolate and pethidine to 10 infants who received glycopyrrolate, alfentanil and suxamethonium. The addition of a muscle relaxant decreased both the duration of hypoxemia and the duration of the procedure by half. Only experienced physicians performed the intubations, which limits generalizability. The durations of procedure and hypoxemia in our morphine group were similar to their pethidine group. Buthada [13] compared thiopenthal in anesthetic doses to placebo in infants over 2 kg. Intubations were shorter and heart rate and blood pressure were more stable in the treatment group. Oei [14] compared morphine, atropine and suxamethonium to awake intubations in 20 infants. Interestingly, even when residents with little or no neonatal experience performed the intubation, the duration of the procedure was significantly shorter and the number of attempts halved in the premedicated group. Barrington [17] used atropine, fentanyl and succinylcholine in 269 consecutive intubations and reported no major complication; no data was available on duration of procedure or vital signs stability. Few very small infants were enrolled in these trials and most used a combination of premedication, which makes comparison with our trial difficult.

Our study has several limitations. First, our sample size is relatively small, which precludes us from eliminating a type 2 error. We began this project with the assumption that severe hypoxemia would occur for about 30 seconds, which was not the case. An observational study would have been useful before making this assumption. Although limited in size, results of this trial should be useful for future investigators and clinicians in their choice of premedication. Second, due to limited resources (unfunded trial), as this study was planned as a pilot, to assess feasibility and adequacy of equipment to obtain data, we decided not to stratify for gestational age. This could have been very useful in refining the findings, as more immature infants may respond differently to premedication in general and have less strength to struggle during an unpremedicated or not sufficiently premedicated painful procedure. Third, as we wanted to mimic our actual NICU practices, in view of modifying such practices, we did not restrict the study intubations to very experienced operators. As a result, there was substantial variability in the level of experience between individuals. Given our small number of patients, this might have impacted on the outcomes. Fourth, several eligible infants were not enrolled. This was due to unavailability of either trial investigators or parents, as many intubations occurred at night. The infants enrolled and those not enrolled had similar birth weights, gestation and reason for intubation, which is reassuring, but does not eliminate the potential for enrolment bias.

## Conclusions

Infants are entitled to effective pain management strategies [18]. It seems only humane to premedicate infants when possible, for known stressful and painful procedures, as we would for older children and adults. Overall, our findings suggest that morphine probably is not the analgesic of choice or insufficient on its own for elective endotracheal intubations. A more rapid onset analgesic like remifentanil, along with atropine should be evaluated and the role of muscle relaxants needs to be better defined. Infants should be stratified either by gestational age or birth weight, to capture differences in their response to premedication and to intubation. Objective measures of pain like the Premature Infant Pain Profile (PIPP) score [19] and/or endocrine indicators of stress should be included in outcomes, which should remain focused primarily on the short-term comfort of the infants but also their safety. Long-term physiologic and clinical outcomes should be incorporated into the trial design. Consideration should be given to including various levels of experience of intubators to increase generalizability and applicability of the findings to units where residents and other allied health professionals are trained to intubated infants.

## Abbreviations

HR heart rate, BP blood pressure, Sp0_2 _oxygen saturation, ETT endotracheal tube, IVH intraventricular hemorrhage, PVL periventricular leukomalacia, NICU Neonatal Intensive Care Unit, PEEP positive end-expiratory pressure

## Competing interests

The authors declare that they have no competing interests.

## Authors' contributions

BL led the study design and manuscript preparation and contributed to data collection. MM contributed to the study design and manuscript preparation. JD contributed to the study design, manuscript preparation and data collection. AK contributed to data gathering and manuscript editing. SG prepared the study medication and contributed her pharmaceutical expertise to the choice of medication dose for the study. All authors read and approved the final manuscript.

## Pre-publication history

The pre-publication history for this paper can be accessed here:


